# Patient reported preferences for sleep interventions among women receiving buprenorphine for opioid use disorder

**DOI:** 10.3389/fpsyt.2023.1244156

**Published:** 2023-09-14

**Authors:** Michelle Eglovitch, Anna Beth Parlier-Ahmad, Catherine Legge, Sajanee Chithranjan, Saisriya Kolli, Stephanie Violante, Joseph M. Dzierzewski, Andrew Stephen Huhn, Allison Wilkerson, Caitlin Eileen Martin

**Affiliations:** ^1^Department of Psychology, Virginia Commonwealth University, Richmond, VA, United States; ^2^School of Medicine, Virginia Commonwealth University, Richmond, VA, United States; ^3^National Sleep Foundation, Washington, DC, United States; ^4^School of Medicine, Johns Hopkins University, Baltimore, MD, United States; ^5^Department of Psychiatry and Behavioral Sciences, Medical University of South Carolina, Charleston, SC, United States; ^6^Institute for Drug and Alcohol Studies, Virginia Commonwealth University, Richmond, VA, United States; ^7^Department of Obstetrics and Gynecology, School of Medicine, Virginia Commonwealth University, Richmond, VA, United States

**Keywords:** insomnia, sleep, buprenorphine, opioid use disorder, substance use disorder treatment

## Abstract

**Aim:**

Among individuals receiving medication for OUD (MOUD), insomnia is highly prevalent and increases the risk for negative OUD outcomes. However, little is known about MOUD patient-reported preferences for insomnia treatments among women with OUD. This mixed-methods study explored acceptability of and patient preferences for sleep interventions among women in OUD treatment.

**Methods:**

This is an analysis from an ongoing cross-sectional survey and interview study investigating the relationship between sleep and OUD recovery. The parent study is actively enrolling non-pregnant women between 18–45 years stabilized on buprenorphine from an outpatient program. Participants complete measures including the Insomnia Severity Index (ISI), with scores of ≥10 identifying clinically significant insomnia symptoms. A sub-sample who met this threshold completed semi-structured interviews. Descriptive statistics were generated for survey responses, and applied thematic analysis was used for interview data.

**Results:**

Participants selected for the qualitative interview (*n* = 11) highlighted prior positive and negative experiences with sleep treatments, challenges with employing non-pharmacological sleep strategies, and preferences for both medical and behavioral sleep interventions while in recovery. Women emphasized the need for flexibility of sleep therapy sessions to align with ongoing social determinants (e.g., caregiving responsibilities) as well as for sleep medications without sedating effects nor risk of dependency.

**Conclusions:**

Many women receiving MOUD have concomitant insomnia symptoms, and desire availability of both pharmacologic and behavioral sleep interventions within the OUD treatment setting. Qualitative findings underscore the need for evidence-based sleep interventions that account for the unique socioenvironmental factors that may impact strategy implementation in this population.

## Introduction

The overdose crisis continues to be a serious public health concern, largely due to opioid-related deaths, which increased 345% between 2001 and 2016 (1, 2). Opioid use disorder (OUD) remains a leading cause of morbidity and mortality. Medication for opioid use disorder (MOUD) is a life-saving treatment shown to reduce overdose risk and improve health outcomes (3). However, heterogeneity in MOUD outcomes persist and discontinuation rates remain high, at least in part due to social determinants of health and comorbid health conditions, underscoring the urgent need for evidence to guide improvements in the quality of OUD treatments.

Among individuals receiving MOUD, sleep disturbances, such as insomnia, are highly prevalent and likely increase risk for substance use recurrence and other negative recovery outcomes, such as reduced quality of life (4, 5). Current prevalence estimates of sleep disorders in people on MOUD range from 19 to 41% (6). Emerging evidence suggests multiple underlying mechanisms of this bi-directional relationship between sleep and OUD treatment trajectories, including increased impulsivity, heightened stress response, chronic pain, and persistent cravings. Additionally, sleep disturbance may interfere with learning during behavioral therapies, compromising patients' abilities to gain optimal treatment benefits (7, 8). Targeting insomnia and sleep dysfunction may be a promising avenue to improve the quality of treatment for OUD.

Importantly, the relationship between sleep disturbance and OUD outcomes may differ by sex, and although OUD remains more prevalent among men, this gap by sex is rapidly closing (9). Specifically, sleep disorders, especially insomnia, are more prevalent among women than men, and one meta-analysis found that females had a risk ratio of 1.41 of insomnia compared to men (10). Proposed underlying mechanisms for this sleep disparity are numerous, ranging from the role of sex-specific hormones to the impact of gendered social factors that more commonly affect women (11, 12). Therefore, the relationship between sleep and OUD is likely multifaceted with interactions with sex and other biopsychosocial factors (8, 9).

Regarding treatments for insomnia, defined as difficulty initiating or maintaining sleep, current options include pharmacotherapy and behavioral approaches. In the general population, common prescription medications that treat insomnia include benzodiazepines, z-drugs, sedating anti-depressants, and other sedating agents, such as anti-epilepsy medications or atypical antipsychotics (13). However, such medications, particularly benzodiazepines, carry a potential for misuse and respiratory depression in populations with OUD. Additionally, people using substances have historically been excluded from insomnia pharmacotherapy trials, leading to a dearth of evidence on their efficacy in this patient population. Despite these risks and limited understanding of the role insomnia pharmacotherapies could play in people in MOUD treatment, prescribing rates are alarmingly high, especially for females (14). A recent study of multi-state insurance claims found that over 70% of OUD patients with insomnia received benzodiazepine prescriptions during the 60 days following buprenorphine initiation; after adjusting for covariates, female sex remained associated with a greater risk of benzodiazepine prescriptions than male sex (14).

Cognitive behavioral therapy for insomnia (CBTi) is currently the frontline evidence-based behavioral treatment for insomnia (15). CBTi, a multi-component approach covering topics such as cognitive restructuring, stimulus control, sleep restriction, and relaxation, has demonstrated efficacy in the general population as well as in clinical populations when adapted to patient's needs (16–19). However, patients' beliefs about sleep problems and practical barriers (e.g., time constraints, work schedules, childcare) can interfere with treatment success (20). Among people with OUD who have more unique barriers to care, there is limited research on CBTi's efficacy. CBTi has demonstrated improvements in sleep primarily among patients with alcohol use disorder (21, 22). To our knowledge, only a single published clinical trial has investigated CBTi among people with OUD; participants included men receiving methadone in Iran, and small improvements in subjective sleep quality were reported (23). Thus, the utility of CBTi as an adjunct to MOUD with buprenorphine for women with comorbid insomnia and OUD is unknown.

Given the complexities lying at the intersection of OUD, sleep, sex, and social determinants of health, it is imperative that insomnia interventions for this patient population are personalized to individuals' biopsychosocial profiles. Further, the unique factors affecting the ability of this population to utilize current medication and behavioral approaches highlights the need for tailored, patient-centered interventions in this domain. Patient insights into what types of interventions are helpful for them, and reasons why, are critical to developing interventions and optimizing their chances of success. However, there is a dearth of research of patient perspectives on sleep interventions for women receiving MOUD. Thus, we conducted an exploratory study to address this gap in the literature and begin to answer the question “what types of sleep interventions would be helpful and usable for women receiving MOUD?” Specifically, the objectives of this study were to (1) describe past experiences and beliefs about sleep and sleep interventions (pharmacotherapy and behavioral), and (2) investigate patient preferences and acceptability of sleep interventions among a sample of women with insomnia receiving buprenorphine for OUD.

## Materials and methods

### Setting and design

This study used a mixed-methods exploratory design to investigate sleep from the perspective of women in recovery from OUD in an outpatient addiction treatment clinic. The outpatient treatment clinic is affiliated with a large academic medical center in a Southern Medicaid-expanded state, which serves as a safety net for the region and treats predominately individuals with low incomes, with many identifying as a minoritized race or ethnicity. The treatment center prioritizes a low-threshold, harm reduction approach, meaning that established patients with recurrence of substance use are not exited from treatment but instead first provided with increased wrap-around support and referral to a higher level of care. Patients in the program are at various stages of recovery and utilize different combinations of services available at the clinic based upon their needs.

### Participants

A research assistant approached patients in the clinic, confirmed eligibility, and invited eligible patients to participate in the study. Patients were pre-identified using a limited medical record review. Study inclusion criteria included the following: OUD diagnosis (24) female sex, stabilized on buprenorphine for at least 6 weeks, aged 18–45, and not pregnant or within 6 weeks postpartum. Exclusion criteria were severe psychosis and non-English-speaking. The Institutional Review Board (IRB) approved all the study components, and informed consent was obtained from all study participants (*N* = 61). Quantitative data were collected via medical record abstraction and an electronic survey, completed either in-person on a provided tablet or remotely on personal electronic device. For current study quantitative analyses, only those who endorsed clinically significant insomnia symptoms were included (*n* = 39). Additionally, those participants who endorsed clinically significant insomnia symptoms were eligible to provide qualitative data by participating in semi-structured interviews. A subsample (*n* = 11) of those with insomnia symptoms completed the interview for the current study.

### Measures

#### Quantitative

Survey demographic items included gender, age, race, employment, education, marital status, and living arrangement. Psychosocial items assessing food, utility, and housing insecurity, healthcare access, and intimate partner violence (IPV) were asked in reference to the past 12 months. Specifically, participants were asked “In the last 12 months, did you ever eat less than you felt you should because there wasn't enough money for food?” and “Are you worried that in the next 2 months, you may not have stable housing?”, to ascertain food and housing insecurity, respectively. Transportation and childcare barriers were assessed using the questions, ‘In the last 12 months, have you ever had to go without healthcare because you didn't have a way to get there?” and “Do problems getting childcare make it difficult for you to work or study?”

The Dysfunctional Beliefs and Attitudes about Sleep (DBAS) Short Form was used to evaluate negative sleep cognitions (25). The DBAS is a 16-item measure that uses a 10-point Likert scale (1- strongly disagree to 10- strongly agree); item scores are averaged for a mean total score ranging from 0 to 10, with higher scores indicating higher endorsement of negative sleep-related cognitions. In addition, the Insomnia Severity Index (ISI) was used to assess insomnia symptoms (26). The ISI is a 7-item measure that uses a 5-point Likert scale (0- none to 4- very severe); items are summed for a total score ranging from 0 to 28, with higher scores indicating higher levels of insomnia symptomatology. Cut-offs of ≥10 were used to identify clinically significant insomnia (26).

The General Anxiety Disorder-7 (GAD-7) was used to assess current symptoms of anxiety. The GAD-7 is a 7-item measure that uses a 4-point Likert scale (0- not at all to 3- nearly every day) in response to questions about frequency of anxiety symptomatology in the past 2 weeks. Item scores are summed for a total score ranging from 0 to 21, with higher scores indicating higher levels of anxiety. Scores ≥10 were considered a positive screen, indicative of clinically significant symptoms of anxiety (27).

The Patient Health Questionnaire-9 (PHQ-9) was used to assess current symptoms of depression. The PHQ-9 is a 9-item measure that uses a 4-point Likert scale (0- not at all to 3- nearly every day) in response to questions about frequency of depression symptomatology in the past 2 weeks. Item scores are summed for a total score ranging from 0 to 27, with higher scores indicating higher levels of depression. Scores ≥10 were considered a positive screen, indicative of clinically significant symptoms of depression (28).

#### Qualitative

A multidisciplinary team (addiction physician, psychologist, sleep expert) generated the interview guide, which utilized open ended questions to explore patient experiences with and preferences for sleep treatments, adapted from the guide used by Rottapel and colleagues (18). Women responded to questions about familiarity with sleep medications and behavioral treatments, and their experiences with such strategies. During the interview, the research coordinator shared a list of CBT-I strategies around sleep hygiene, stimulus control, and cognitive restructuring, and explored experiences and anticipated challenges with implementing such strategies. Finally, women were asked to think about a tailored sleep health program and respond about barriers and facilitators to participating in such a program. Participants were compensated $15 for their time.

All participants completed interviews using Zoom, a virtual conferencing platform, or in-person in a private office. Two study team members conducted the interviews, with the research coordinator administering the interview and a research assistant taking notes during interviews. Interviews were audio-recorded and lasted 30–60 min. The interviews were transcribed using Zoom transcription software, with the research assistant listening to the transcript and checking the Zoom-automated transcript for fidelity and editing as needed for clarity.

### Data management and analysis

Descriptive statistics were generated for demographic, clinical, and psychosocial characteristics as well as self-reported sleep attitudes for the sample with clinically meaningful insomnia symptoms and the qualitative sub-sample.

We utilized the concept of information power, which posits that when powerful data are obtained from each individual participant, then a small sample size may be adequate. Interviews were conducted until data saturation was reached and information redundancy occurred, meaning when new interviews were being conducted no new information was obtained (29, 30).

The interview data were analyzed using a qualitative content analysis approach. This approach allowed for participants' data to provide a comprehensive description of their individual experiences, facilitating a greater understanding of the importance of and beliefs about sleep and sleep interventions for women in treatment for OUD. The qualitative content analysis method facilitated the determination of themes related to the participants' experiences.

The analysis process consisted of several steps. Once interviews from Zoom were transcribed and finalized in Microsoft Word, the analysis team [PI (CEM), research coordinator (ME), and 3 research assistants (CL, SC, SK)] individually reviewed each transcript line by line. The same authors then re-read the transcripts and assigned codes to key concepts that arose from the data. The team met on Zoom to discuss topics that emerged from the codes and, using an inductive process, created a category code list to organize the qualitative data (31). Through group consensus, the group condensed the list into the most salient codes. Finally, an iterative process was then used to group codes and finalize themes and subthemes. The authors identified quotes to highlight themes.

## Results

### Quantitative

Thirty-nine participants (63.9% of the total study sample) endorsed clinically significant insomnia symptoms, of which 11 participated in a qualitative interview (Table 1). Demographic and clinical characteristics of the qualitative subsample generally reflected those of the larger sample of study participants with clinically elevated levels of insomnia symptoms. Among the 11 participants who completed the qualitative interview, 9 (81.8%) of participants were white, and 2 (18.2%) were Black. The mean age of the qualitative participants was 35.2 (SD = 5.9) years. Five participants (45.5%) indicated that they were currently using medication for sleep, and 2 participants (18.2%) indicated currently receiving therapy for sleep. The average length of time in OUD treatment was 24 months (range: 75–60 months). The most common substances participants reported past 28-day use were for nicotine (*n* = 7) and cannabis (*n* = 5).

**Table 1:** Sociodemographic characteristics of participants.

**Characteristic**	**Clinically significant insomnia symptoms *N* (%) *n* = 39**	**Qualitative participants *N* (%) *n* = 11**
**Demographics**		
**Age [M (SD)]**	36.3 (9.0)	35.2 (5.9)
**Race**		
White	22 (56.4%)	9 (81.8%)
Black	14 (35.9%)	2 (18.2%)
**Employment**		
Unemployed	21 (53.8%)	8 (72.7%)
Employed	11 (28.2%)	3 (27.3%)
**Education**		
<High School	13 (33.3%)	5 (45.5%)
> High school	26 (66.7%)	6 (54.5%)
**Marital status**		
Unmarried	21 (52.8%)	7 (63.6%)
Married	18 (46.2%)	4 (36.4%)
**Current sleep treatment behaviors**		
Using medication for sleep (% yes)	20 (51.3%)	5 (45.5%)
Receiving therapy for sleep (% yes)	4 (10.3%)	2 (18.2%)
**Mental health symptoms**		
Clinically significant anxiety (% yes)	21 (53.8%)	4 (36.4%)
Clinically significant depression (% yes)	29 (74.4%)	6 (54.5%)
GAD-7 score (range: 0–21)	10.9 (6.4)	8.9 (4.9)
PHQ-9 score (range: 0–27)	14.3 (6.6)	12.1 (6.7)
**Social determinants of health**		
Food insecure (% yes)	19 (48.7%)	5 (45.5%)
Utilities insecurity (% yes)	6 (15.4%)	0 (0.0%)
Housing insecure (% yes)	17 (43.6%)	5 (45.5%)
Challenges affording healthcare (% yes)	8 (20.5%)	2 (18.2%)
Lack of transportation to healthcare (% yes)	12 (30.8%)	4 (36.4%)
Healthcare literacy challenges (% yes)	5 (12.8%)	0 (0.0%)
Lack of childcare (% yes)	13 (33.3%)	3 (27.3%)
Fear of current partner (% yes)	6 (15.4%)	1 (9.1%)

Participants commonly endorsed challenges with social determinants of health (Table 1). Almost one half of participants endorsed food and housing insecurity, and approximately one third endorsed transportation and childcare barriers. In addition, approximately one fifth of participants indicated challenges affording healthcare.

**Figure 1:**
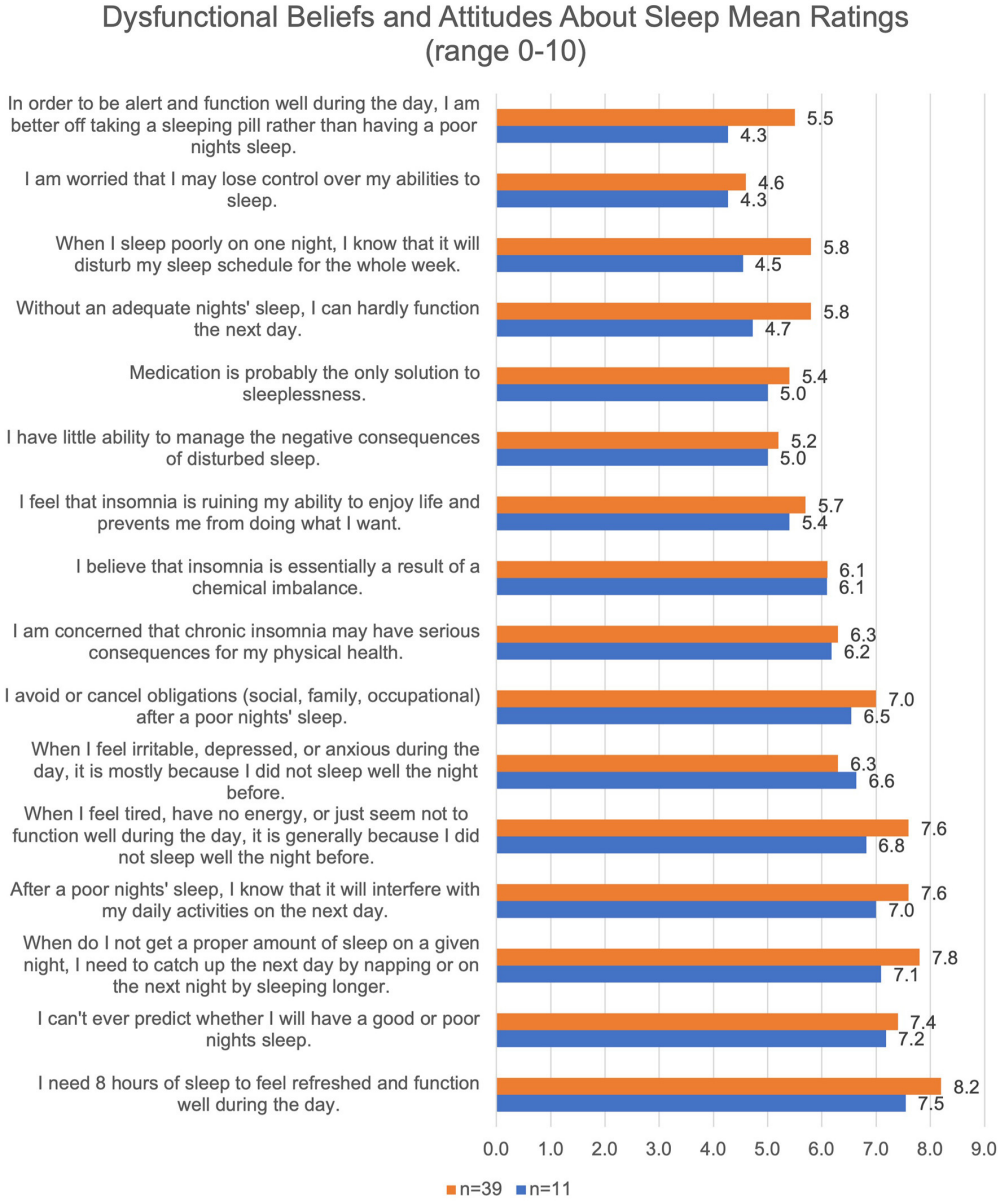
Negative Sleep-Related Cognitions of Participants.

Participants commonly endorsed negative sleep-related cognitions on the DBAS. Levels of such cognitions were similar between the overall sample of participants clinically elevated levels of insomnia symptoms (*M* = 5.9, SD = 1.1) and the qualitative sub-sample (*M* = 6.4, SD = 1.1). Many qualitative participants endorsed cognitions about poor sleep interfering with their daily functioning and needing to “catch up” on sleep when they do not get the proper amount. The most highly endorsed cognition was “I need 8 h of sleep to feel refreshed and function well the next day” (Figure 1). The least commonly endorsed cognition amongst qualitative participants was “In order to be alert and function well during the day, I am better off taking a sleeping pill rather than having a poor nights' sleep”.

### Qualitative

Four themes emerged from the interviews: (1) positive and negative experiences with sleep treatments, (2) preferences for medications to improve sleep while in OUD recovery, (3) challenges to employing non-pharmacological strategies intended to improve sleep, and (4) preferences for therapy intended to address sleep for individuals in OUD treatment. Table 2 provides a list of the themes, sub-themes within each respective theme, and a representative quote.

**Table 2:** Qualitative themes, codes and quotes.

**Theme**	**Sub-theme**	**Quotation**
Prior experiences with sleep treatments	Medications that have negatively and/or positively impacted sleep	“In recovery, the hydroxyzine does help with the anxiety and does help get me to sleep. I am not so anxious when I do try to go to sleep. But that's about it for now during recovery, I try to stay away from anything.”—(Study ID 57)
	Medication and behavioral sleep treatments are typically siloed	“I feel like I never have taken the time to really focus on [sleep]. I've just fixed it with a pill. And I'm not blaming the doctors, and I'm not saying they're just throwing medication at it. But when they're seeing 5,000 patients a week, what more do they have to offer when they see you once a every 6 months to a year. I feel like if you took therapy and sleep therapy and medication together, it would probably really work.”—(Study ID 34)
Preferences for medications to improve sleep while in OUD recovery	Ability to improve sleep quality and quantity	“[I would take a medication for sleep because] I would love to know what it feels like to sleep for like six hours straight or even four. I would take it if I knew I could stay asleep more than three to four hours straight.”—(Study ID 47)
	Beneficial to mood	“I would know a medication for sleep is working because I would notice waking up, you know, rested and feel better.”—(Study ID 45)
	Minimal sedation effects	“I would be willing to try [a sleep medication] because I would definitely like to be sleeping better. It also depends on what it is, because I still have to be able to wake up if my daughter is crying or if something happens.”—(Study ID 21)
	No risk of developing dependence on the medication	“[I would be interested in a sleep medication] as long as it's not a narcotic or a benzo or any of those. I just don't want to feel like that ever again. Because I feel like, if I were to get something that were to give me that feeling again. I hate to say it even though it has been this long. I'm so scared that I'lI just take right to it. And I don't want to take something from my doctors that gives me that feeling and then I take it to the street and end up relapsing over it. I think that's my biggest fear.”—(Study ID 34)
Challenges to employing non-pharmacologic strategies for sleep while in OUD recovery	Abstaining from substances that interfere with sleep is challenging	“Nicotine would be hard to stop, because I smoke cigarettes, and also the tea because I like tea. I'm trying to switch to decaf now and I drink coffee in the morning. But I don't know, when I first stopped using and drinking, it was a lot of coffee all the time, so it was like a pleasure so now I'm just in the habit of coffee or tea now.”—(Study ID 22)
	Mental health symptoms can interfere with implementation of behavioral strategies for sleep	“If I can't get to sleep and then I'm tossing and turning and then looking at the clock. I know you're not supposed to be looking at the clock. And then I'll go and be like I'm not looking at the clock, I'm not, I'm not, and then next thing I'm looking at it and then I only have so many hours left to sleep and all this and tomorrow's going to be a shitty day. Woo-hoo that's me!”—(Study ID 14)
	Current physical environment renders behavioral strategies hard to implement	“[Reducing time spent awake in bed] Just because my room is the only place that I've got that is, like, quiet and away from everybody else. Because there's twelve people in the house, like eight kids and four adults.”—(Study ID 41)
Preferences for non-pharmacologic sleep treatment delivery method for individuals in OUD treatment	Convenience of telehealth options due to ongoing social determinants as barriers to in-person sessions	“Getting to in-person sessions would be hard because my car messes up a lot. Like, just always need stuff done with it.”—(Study ID 71)
	Need for flexibility in session durations and formats to accommodate competing schedules and responsibilities	“Like you know if it's more convenient for me to be at home to do it versus me doing the commute. I do live at home with my parents and my grandma has a mother-in-law suite here too, after I do this today, I have to do something for her so it's kind of convenient if we've got more than one thing going on during the day. Because that commute, that's like an hour and a half out of the day that something else could have been done.”—(Study ID 14)
	In-person options promote treatment accountability	“[In-person therapy] holds yourself accountable to be 100% honest and open on what's really going on. There is a lot you can tell from a person… I speak with my hands, my facial expression, how I'm carrying myself. So, going in person would probably make me feel a lot more believable to the process, than just being told over the phone or over video “hey do this, and tell me how it goes.”—(Study ID 34)

#### Theme 1: Prior experiences with sleep treatments

Generally, participants expressed both positive and negative experiences with treatments for sleep. The most commonly mentioned medications were zolpidem (*n* = 4), trazodone (*n* = 4), and quetiapine (*n* = 3). Some medications, such as zolpidem and hydroxyzine were explicitly prescribed to participants to assist with sleep. However, participants also shared experiences with over-the-counter medications, such as “Tylenol PM” and melatonin supplements, to help with sleep. Participants had mixed experiences with medication and impact on sleep. For example, two participants indicated that trazodone helped with sleep while two others noted it was not particularly helpful with sleep.

Participants also shared experiences with behavioral strategies. They were familiar with behavioral strategies for sleep and reported being familiar with sleep hygiene strategies such as reducing electronic usage before bed and limiting screens (e.g., television) in the bedroom (*n* = 5), meditation and mindfulness (*n* = 4), and stimulus control, limiting the bed only to sleep (*n* = 2). Participants reported that behavioral and medication treatments are often siloed and delivered by different healthcare providers and entities, making the implementation of such strategies more challenging.

#### Theme 2: Preferences for medications to improve sleep while in OUD recovery

All participants (*n* = 11) expressed that they would take a medication to improve sleep if it was prescribed by their health care provider. They noted a preference for a safe medication to improve sleep quality and quantity, particularly to lengthen the duration of sleep. Participants expressed the desire for a medication to have minimal side effects, such as grogginess the following morning, and improve irritability, as lack of sleep would often affect their mood negatively. However, because most of the sample were caregivers for children or family members (*n* = 10), they reported that the medication could not “knock them out” or be too sedating that they would not be able to give care in the middle of the night. In addition, participants expressed that the medication could not be associated with risk of dependence. Participants shared that recovery was important to them, and any medication that would interfere with their recovery was undesirable.

#### Theme 3: Challenges to employing non-pharmacologic strategies for sleep while in OUD recovery

Most participants had heard of non-pharmacologic sleep strategies. However, participants expressed that these strategies, among others, are hard to employ for various reasons. For example, the majority of participants expressed regular consumption of caffeine and nicotine, two substances that negatively impact sleep. Participants also shared that their physical environments would make implementation of such strategies difficult, as they reported challenges with sharing small spaces with multiple people, or sleeping in different areas of the house so that they could be close to family members to give care.

Participants also expressed that negative cognitions that occur at night impact the ability to engage in behavioral strategies. For example, several participants noted that “stinking thinking”, a concept referring to negative cognitive distortions about ability to maintain recovery as well as negative memories associated with active addiction are common at night and while trying to initiate sleep, worsening insomnia symptoms.

#### Theme 4: Preferences for non-pharmacologic sleep treatment delivery method for individuals in OUD treatment

Participants noted pros and cons of both telehealth and in-person delivery options for therapy to address issues with sleep. Most participants noted that they appreciated telehealth options, particularly due to the convenience of being able to do sessions from home. However, some participants also reflected that telehealth has challenges, specifically lack of phone or internet connection required to complete telehealth sessions depending on their geographic location. Participants expressed that in-person sessions would keep them accountable to the process of behavioral sleep treatment, and that accountability is a factor in recovery as well. Conversely, participants also shared that attending in-person sessions can be challenging depending on childcare or transportation needs and reflected that flexibility of having both virtual and in-person options may help address this.

## Discussion

This mixed-methods study explored past experiences and beliefs about treatments (pharmacologic and behavioral) for sleep disturbances as well as preferences and acceptability of sleep interventions among women with comorbid insomnia receiving buprenorphine in outpatient OUD treatment. We utilized quantitative data to describe the sample and supplemented such data with rich qualitative data from in-depth interviews.

Women receiving MOUD described both high quality and appropriate quantity of sleep were important to their daily functioning and their recovery. They highlighted varying impacts of their experiences with medication and behavioral therapy for sleep. However, existing modalities of sleep interventions are largely insufficient in this population for a variety of reasons. For instance, although the women in this sample reported that they would take a medication if prescribed by their provider, most of our sample would rather cope with a poor night of sleep than take a “sleeping pill.” Medications perceived to carry the risk of dependence could compromise their recovery and are undesirable, precluding several sleep medications commonly prescribed for insomnia (32). Clearly many existing pharmacological interventions for insomnia may not be appropriate for this population due to their OUD and associated experiences and recovery goals. Overall, these preferences expressed by women in our sample are important to consider as new pharmacologic insomnia interventions are generated and evaluated in the MOUD patient population.

Women also reported that they would not want a medication that was too sedating, as it would impact their ability to take care of family members. Participants commonly reported that caregiving was a barrier to sleep. Similarly, Rottapel et al. found in their qualitative exploration of barriers to sleep among a community sample that caregiving was cited as the most common barrier. Parents valued sleep, yet the demands of childcare negatively impact sleep (2019). Women experience caregiver strain more than men, which may impact their ability to address their own healthcare needs such as quality sleep.

**Figure 2:**
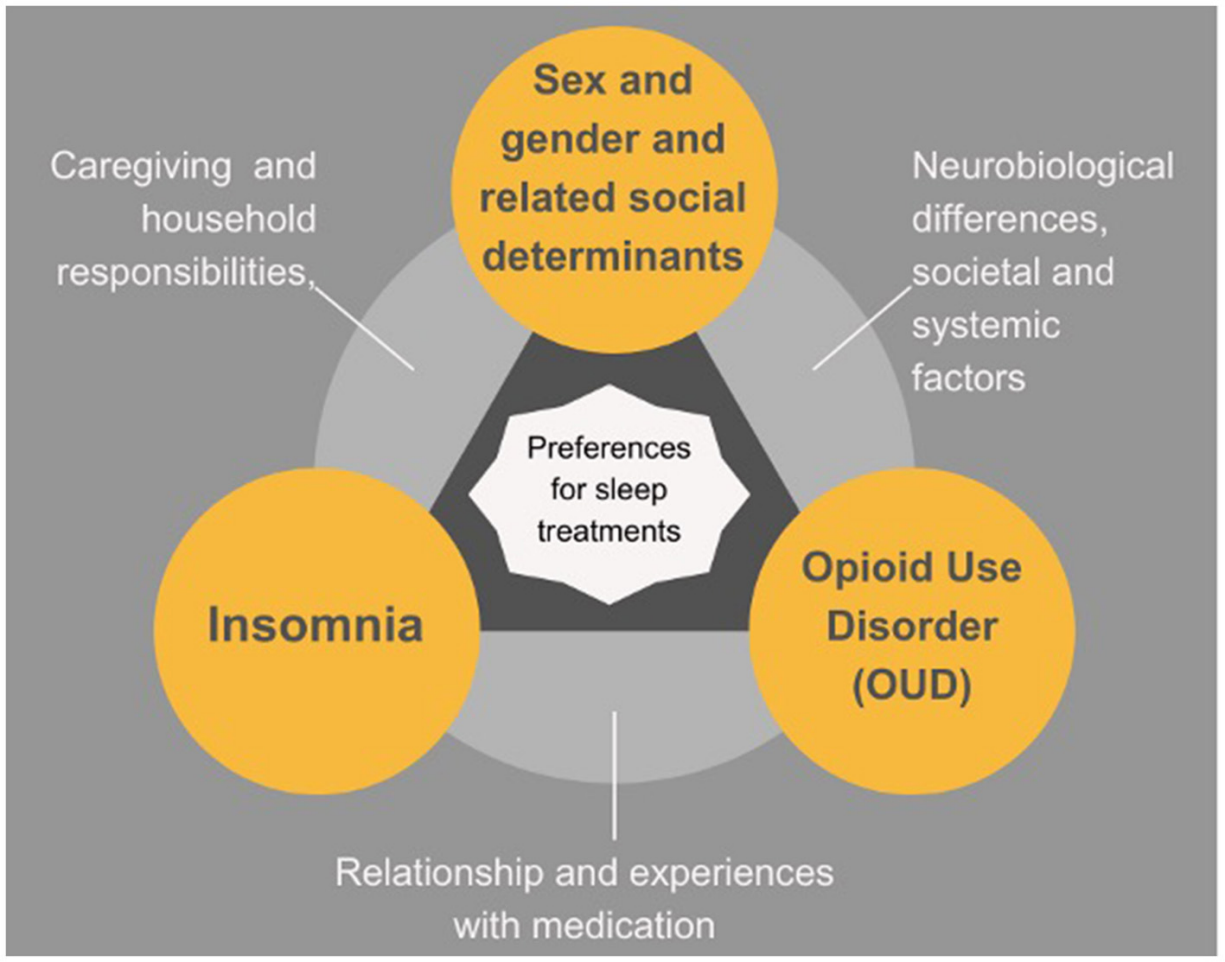
Insomnia in context.

The participants also detailed how social determinants of health can impact successful participation in pharmacologic and non-pharmacological sleep interventions by MOUD patients (conceptualized in Figure 2). For example, transportation barriers were endorsed in both quantitative and qualitative results. Most of the women in our sample indicated that they often had transportation issues that would impede them from being able to attend regular in-person therapy sessions, but also noted internet and phone connectivity issues as barriers to telehealth sessions. In addition, women often noted that in-person sessions help with accountability to the therapeutic process, with accountability being a major component of recovery. Other social determinants of health commonly reported that may affect sleep and ability to participate in in-person interventions, included housing insecurity and challenges obtaining childcare. Thus, the myriad of factors that can impact ability to participate in non-pharmacological treatment necessitate flexible and adaptable modalities of sleep interventions, emphasizing the importance of individualizing treatments for MOUD patients.

Targeted sleep treatment may have impacts not only on insomnia, but also on co-morbid mental health conditions. Almost half of the qualitative sample endorsed clinically significant anxiety and over half the sample endorsed clinically significant depression. Further, participants reflected on their experiences with negative memories associated with active addiction, indicating a need to co-treat broader mental health concerns such as PTSD, anxiety, and depression. Insomnia can have bi-directional relationships with anxiety and depression, and thus treatment of insomnia might alleviate mental health symptoms (33). Thus, targeting insomnia in OUD treatment may be an avenue to simultaneously target multiple underlying mechanisms (e.g., negative emotionality, impulsivity) contributing to MOUD outcomes, an area in need of further investigation.

### Clinical and research implications

Insomnia is prevalent among women receiving MOUD. It impacts quality of life and OUD recovery. Current evidence-based treatments are ill-suited for this population. Findings highlight the need to provide holistic, flexible, integrated insomnia and mental health treatment with addiction treatment. Future studies should address development, implementation, and evaluation of tailored sleep treatments among people with OUD that account for the unique social determinants of health and other sleep-related considerations that affect this population.

### Limitations

We only included women currently in treatment, receiving MOUD at a single outpatient addiction treatment clinic in the U.S., which limits generalizability of findings to other populations or settings. However, our selective sample is also a strength, as our findings are pertinent to an increasingly more prevalent patient population in the overdose crisis. Our findings shed light on sleep in this population, allowing us to learn from their experiences to expand the evidence base to improve sleep treatment for women with OUD.

## Conclusion

Clinically elevated insomnia symptoms were highly prevalent among women with OUD. Findings suggest delivery of existing modalities of sleep interventions are insufficient for this population. This research highlights the need for evidence-based, tailored treatment options that account for the unique social determinants of health and other sleep-related considerations that affect this population. Incorporating patient preferences and input through a sex and gender informed lens into the development, implementation, and evaluation of sleep treatments among people with OUD has the potential to make an immediate and significant positive impact on this population.

## Data availability statement

The raw data supporting the conclusions of this article will be made available by the authors, without undue reservation.

## Ethics statement

The studies involving humans were approved by Virginia Commonwealth University. The studies were conducted in accordance with the local legislation and institutional requirements. The participants provided their written informed consent to participate in this study.

## Author contributions

ME: data collection, analysis, and writing and editing. AP-A: review and editing. CL, SC, and SK: analysis. SV: conceptualization and data collection. JD: conceptualization, design, and review and editing. AH and AW: review and editing. CM: conceptualization, methodology, supervision, writing, review, and funding acquisition. All authors contributed to the article and approved the submitted version.
